# Limb Strength and Power Asymmetries in Professional Team Sport Athletes at Return-to-Sport Testing Following ACL Reconstruction

**DOI:** 10.3390/medicina62040654

**Published:** 2026-03-29

**Authors:** Marko D. M. Stojanović, Nikola Andrić, Tatjana Jezdimirovic Stojanovic, Šime Veršić, Julio Calleja Gonzalez

**Affiliations:** 1 Faculty of Sport and Physical Education, University of Novi Sad, 21000 Novi Sad, Serbia; nikola.trenaznaekspertiza18@gmail.com; 2 Training Expertise Laboratory, 21000 Novi Sad, Serbia; tatjana.stojanovic@fzs.edu.rs; 3 Faculty of Sport, Union-Nikola Tesla University, 11000 Belgrade, Serbia; 4 Faculty of Kinesiology, University of Split, 21000 Split, Croatia; sime.versic@kifst.eu; 5 Department of Physical Education and Sports, Faculty of Education and Sport, University of the Basque Country (UPV/EHU), 01006 Vitoria-Gasteiz, Spain; julio.calleja.gonzalez@gmail.com

**Keywords:** isokinetic dynamometry, countermovement jump limb symmetry index, deceleration, concentric power, eccentric power

## Abstract

*Background and Objectives*: Present assessment methods have not effectively mitigated the risk of recurrent anterior cruciate ligament (ACL) injury following reconstruction (ACLR), suggesting that critical neuromuscular deficits may be underdiagnosed. This study aimed to compare limb asymmetries across strength, concentric and eccentric power, and deceleration metrics during return-to-sport (RTS) testing in professional athletes post-ACLR. *Materials and Methods*: Forty-four participants (33 males, 11 females; age 22.5 ± 5.8 years, body mass 75.9 ± 13.0 kg, height 180.5 ± 8.38 cm) (mean ± SD) with a unilateral reconstructed ACL (BTB = 33, HT = 11 graft) were included. They underwent isokinetic testing of knee flexor and extensor strength and bilateral countermovement jump (CMJ) assessments to measure concentric and eccentric peak power and deceleration metrics. Limb symmetry indices (LSI) were calculated for each parameter. Welch’s ANOVA and Games–Howell post hoc tests were used to compare LSIs among parameters. *Results*: Welch’s ANOVA showed that limb symmetry differed significantly across the measured neuromuscular parameters (F = 12,59, *p* < 0.001). Knee flexor strength LSI was significantly higher than knee extensor strength LSI (*p* = 0.003; d = 1.18), concentric peak power LSI (*p* < 0.001, d = 1.44), eccentric peak power LSI (*p* = 0.001, d = 1.71), and deceleration LSI (*p* = 0.001, d = 2.09). In addition, deceleration LSI was significantly lower than knee extensor strength LSI (*p* = 0.001, d = 1.34) and concentric peak power LSI (*p* = 0.007, d = 1.10). No significant difference was found between concentric and eccentric peak power, nor between knee extensor strength and either concentric or eccentric peak power LSIs. *Conclusions:* The findings of this study revealed significantly greater asymmetries in load absorption capacity compared to strength and concentric power measures at return-to-sport time frame in professional athletes post-ACLR.

## 1. Introduction

Anterior cruciate ligament (ACL) injuries are one of the most common and severe knee injuries encountered in team sports, often requiring surgical intervention and extensive rehabilitation [1]. While several surgical techniques exist to address ACL rupture, including internal brace reconstruction, the majority of evidence supports standard ACL reconstruction as the primary treatment modality [2]. The primary goals of ACL reconstruction and subsequent rehabilitation are to restore knee stability, rebuild neuromuscular capacity and enable a safe return to pre-injury levels of sport participation. Despite advances in surgical techniques and rehabilitation protocols, athletes experience persistent neuromuscular deficits long after surgery, placing them at increased risk for secondary ACL injuries and compromised performance [3,4], with prevalence of reinjury ranging between 9% and 29% [1] To mitigate these risks, return-to-sport (RTS) criteria comprising objective test batteries have been developed. These aim to assess athletes’ readiness to safely resume sport, minimize re-injury risk, and restore pre-injury performance [5,6]. However, actual return rates remain suboptimal: only 55% of individuals return to competitive sport following ACL reconstruction [7], with many elite athletes reducing participation or retiring within three years of surgery [4,8]. These concerning rates suggest that some additional neuromuscular deficits—beyond those reported by standard RTS tests—may persist and contribute to these adverse outcomes [5].

Muscle strength deficits following ACL reconstruction have been widely documented as an important return-to-sport criterion, particularly in the quadriceps and hamstring muscle groups [9]. These strength deficits are commonly assessed using isokinetic dynamometry, which measures peak torque of the knee extensors and flexors [10]. Reduced concentric muscle peak torque after ACL reconstruction has been associated with movement compensations during high-load activities such as jumping and hopping [10], inferior subjective and objective knee function [11], and an elevated risk of knee re-injury [1]. Current recommendations suggest that muscle strength measured by isokinetic dynamometry presents a standard and essential assessment tool in the RTS test battery [12], with the widely accepted benchmark now requiring patients to achieve at least 90% strength symmetry compared to the uninjured limb before clearance for RTS [13,14].

Beyond strength, growing evidence supports the critical role of power-related parameters as complementary—and sometimes superior—indicators of functional readiness for return to sport [5]. Power reflects the ability to generate force rapidly and is crucial for dynamic sport-specific tasks such as jumping, sprinting, and cutting [15,16], impacting both performance and injury resilience [17]. Deficits in power output can persist even when strength values approach normal levels, potentially masking underlying neuromuscular impairments that compromise movement mechanics and predispose athletes to re-injury [18].

Advancements in wearable technology and force platforms have enhanced the assessment of power performance, with the countermovement jump (CMJ) emerging as a widely used test in return-to-sport evaluations [19]. Beyond concentric power, eccentric power parameters have recently garnered recognition for their vital role in return to sport assessment after ACL rehabilitation [20]. Eccentric muscle actions are crucial for absorbing load, decelerating the body, and stabilizing joints during sport-specific tasks such as landing and cutting—the very phases during which ACL injuries often occur [21]. Deficits in eccentric power compromise shock absorption and neuromuscular control, contributing to both reduced athletic performance and elevated secondary injury risk [22]. Together, concentric and eccentric power metrics provide complementary insights into neuromuscular function that strength testing alone may not capture.

Although CMJ tests can yield various parameters (e.g., peak power, rate of force development, time to peak force), clinical decision-making requires prioritizing those with high reliability, interpretability, and clear links to functional outcomes. Peak power remains a cornerstone due to its superior reliability, ease of measurement, and strong association with functional performance and injury risk [23,24]. However, incorporating eccentric power parameters—such as peak eccentric power and eccentric deceleration rate—can enhance the understanding of neuromuscular function by capturing aspects of force generation and absorption over time [20]. These eccentric metrics quantify how rapidly the muscles can slow down the body during landing or cutting, a critical component of joint stabilization and injury prevention [25].

Limb asymmetry in muscle function is a well-established risk factor for ACL patients [5]. Persistent differences between the injured and uninjured limbs can adversely alter biomechanics during critical sport-specific movements such as jumping, cutting, and landing. These altered movement patterns may increase joint loading and strain on the reconstructed ACL graft or the contralateral limb, thereby raising the risk of re-injury [25]. Moreover, asymmetries often reflect incomplete neuromuscular recovery, delayed functional adaptation, or compensatory movement strategies that can undermine both injury resilience and performance outcomes. Consequently, identifying and correcting limb asymmetries is a central component of return-to-sport rehabilitation protocols. Asymmetries are usually quantified using the limb symmetry index (LSI), calculated as (value of the injured leg/value of the uninjured leg) × 100% [26].

To date, no study has conducted a direct, comparative analysis of limb asymmetries across muscle strength and concentric and eccentric power parameters within the same cohort of ACL-reconstructed team sport athletes at the return-to-sport stage, to the best of the authors’ knowledge. This represents a significant gap, given that each of these neuromuscular measures reflects distinct physiological and biomechanical functions relevant to sport-specific demands and injury mechanisms [5]. The late-stage rehabilitation (six to nine-months) is a key phase when return-to-sport decisions are commonly made [14,27], yet it remains unclear which neuromuscular deficits persist most strongly at this time and thus may be overlooked by standard clinical assessments. Understanding the relative magnitude of limb asymmetries in these parameters will enable clinicians and researchers to more precisely detect residual impairments, tailor rehabilitation programs, and optimize RTS decision-making to enhance athlete safety and long-term outcomes. Therefore, this study aims to quantify and compare limb asymmetries across five neuromuscular parameters in team sport athletes approximately nine months post-ACL reconstruction. These parameters include concentric muscle strength of the knee flexors and extensors measured using an isokinetic dynamometer, as well as concentric and eccentric power and deceleration outputs during countermovement jumps (CMJ). We hypothesize that eccentric-related metrics will exhibit greater limb asymmetry than concentric power and muscle strength measures at this stage of recovery.

## 2. Materials and Methods

### 2.1. Participants

This retrospective cohort study involved team sport athletes undergoing rehabilitation following anterior cruciate ligament reconstruction (ACLR) who completed return-to-sport testing at our rehabilitation center. The database was queried from March 2021 to April 2025 to identify patients who underwent primary ACLR using patellar tendon or hamstring tendon autografts, and who completed both isokinetic and force plate jump testing. Inclusion criteria required athletes to be over 17 years old and to be professional team sport athletes competing at least at the national level. Patients with any associated meniscal lesion requiring repair or partial meniscectomy, cartilage lesions, or relevant prior surgery on any other joint of the lower limbs were excluded. Additionally, all participants had to have completed a supervised rehabilitation program, verified by their rehabilitation specialist, and been referred for return-to-sport testing by their physician. From an initial pool of 90 patients, 36 were excluded—25 due to failure to meet inclusion criteria and 11 due to incomplete or missing data. The final sample consisted of 44 patients (31 men, 11 women) with characteristics presented in Table 1. The study was approved by the Ethical Committee of the University of Novi Sad (Ref. No. 25-02-04/2025-1), and all patients provided written informed consent prior to participation. For patients under 18 years of age at the time of testing, parental or guardian consent was also obtained.

### 2.2. Study Design

This study employed a within-participant cross-sectional design to identify limb asymmetries in neuromuscular function following ACL reconstruction. All testing measurements were collected in a single visit, at the same time of day (9:00 a.m.–11:00 a.m.) during regular return-to-sport battery tests. Testing procedures were conducted in the Training Expertise Laboratory, Novi Sad, Serbia, supervised by a full professor (M.S.) and one assistant, PhD student (N.A.). Testing sessions were performed under similar environmental conditions for all participants (21 °C and; 60% humidity). The primary outcomes included knee flexor and extensor muscle strength measured via isokinetic dynamometry, and concentric and eccentric power parameters obtained from countermovement jump (CMJ) testing. All participants completed a standardized warm-up lasting 12–15 min, which included cycling, foam rolling, dynamic stretching, low-intensity jogging, bodyweight activation, and landing drills. Testing order was standardized as follows: (a) anthropometric measurements, (b) CMJ power testing, and (c) isokinetic strength assessment. Each testing session lasted approximately 60–75 min, during which participants were familiarized with all procedures through verbal explanation and live demonstration by the same research personnel to ensure consistency. Participants were instructed to refrain from strenuous physical activity for 48 h prior to testing to minimize performance variability. No adverse events or injuries occurred during testing. Limb symmetry indices (LSI) were calculated for each neuromuscular parameter to quantify side-to-side differences within participants.

### 2.3. Testing Procedures

#### 2.3.1. Anthropometrics

All anthropometric measurements were performed by an accredited level 2 anthropometrist (M.S.) following the International Society for Advancement of Kinanthropometry guidelines. The athlete’s height was measured to the nearest centimeter using a portable stadiometer with a sliding headpiece (SECA^®^ 213, Hamburg, Germany), while their weight was assessed to the nearest tenth of a kilogram with a SECA^®^ model scale (Seca GmbH, Hamburg, Germany).

#### 2.3.2. Isokinetic Muscle Strength

Isokinetic dynamometry—concentric peak torque of the knee extensors and flexors—was measured at an angular velocity of 60°/s [10] using a Kineo Training System (V7, GLOBUS^®^, Bolzano, Italy). The device was calibrated in accordance with the manufacturer’s specifications. Participants were instructed to perform maximal-effort, maximal-velocity contractions for both knee extension and knee flexion. A 60 s rest interval was provided between legs and a 240 s rest interval between the extension and flexion tasks. Lever length, defined as the distance from the center of the knee joint proximally to the lateral malleolus distally, was used to calculate peak torque. Peak torque was calculated as: Peak torque = Peak force × Lever length × 9.81 m/s^2^ (N·m).

Knee extension—The test begins with participants seated, with hips and knee flexed at 90 degrees. On command from the strength and conditioning coach (S&C), the participant exerts maximal effort to extend the knee fully, then gradually returns to the starting position. Participants performed three trials for each leg; the best performance measure was chosen for further analysis.

Knee flexion—The test starts with the participant standing, with the knee and hip fully extended. In response to the S&C coach’s command, the participant pulls their heel towards their hip with maximal force until reaching a 90-degree angle, then slowly returns to the starting position. Participants performed three trials for each leg; the best performance measure was chosen for further analysis.

#### 2.3.3. Power Test

Countermovement Jump (CMJ)-CMJ was assessed using two synchronized force plates (Delta Force Plate; Kinvent^®^; V2; 2000 Hz; Montpellier, France), allowing for independent measurement of ground reaction forces from each limb, facilitating accurate calculation of the Limb Symmetry Index (LSI). This system has demonstrated good concurrent validity and reliability for vertical jump assessments [28]. Participants started the test in an upright position, then, upon the tester’s signal, transitioned into a self-selected semi-squat position, from which they accelerated into the air, using powerful triple extension. Participants were instructed to always keep their hands on their hips during the test, to reduce the time period between the eccentric and concentric phases of the jump, and to jump as fast and as high as possible. Three trials with a passive pause of 45 s were performed, and the trial with the best jump height was used for further analysis [1]. Three performance measures—concentric peak power, eccentric peak power and deceleration—were calculated for both legs and used for further analysis.

### 2.4. Statistical Analysis

Normality of distributions was assessed for each neuromuscular parameter using the Shapiro–Wilk test, appropriate given the sample size of fewer than 50 participants per group. The Levene test indicated significant heterogeneity of variances across the neuromuscular parameters (*p* < 0.05); therefore, Welch’s heteroscedastic one-way analysis of variance (ANOVA) was employed to compare Limb Symmetry Indices (LSIs) across the five neuromuscular metrics. This test does not assume equal variances among groups. For pairwise comparisons between parameters, Games–Howell post-hoc tests were conducted, which also accommodate unequal variances and sample sizes. The effect sizes for post-hoc pairwise comparisons were computed using Cohen’s d and interpreted according to the guidelines proposed by Hopkins [29]: values between 0.00 and 0.19 were considered trivial, 0.20 to 0.59 small, 0.60 to 1.19 moderate, and those exceeding 1.20 classified as large.

A post hoc power analysis was performed using G*Power 3.1 [30] based on a one-way fixed-effects ANOVA model with five groups, an alpha level (α) of 0.05, an effect size estimate of η^2^ = 0.06 (medium effect), and total sample size (n) of 44 participants. This approach approximates the power of the Welch ANOVA used in the main analysis. The analysis yielded approximately 80% power to detect medium effect sizes under these parameters.

All statistical analyses were performed using IBM SPSS^®^ Statistics version 25 (IBM Corp., Armonk, NY, USA). Statistical significance was set at *p* < 0.05. Additionally, limb deficits for selected parameters were calculated as percentage differences using the formula: Percent Deficit (%) = [(Uninvolved Limb − Involved Limb)/Uninvolved Limb] × 100.

## 3. Results

Table 2 summarizes the mean and standard deviation (SD) values of key neuromuscular variables measured in the injured and non-injured limbs.

Welch’s ANOVA showed a significant effect of parameter type on LSI, demonstrating that limb symmetry differed significantly across the measured neuromuscular parameters (Table 3).

**Figure 1:**
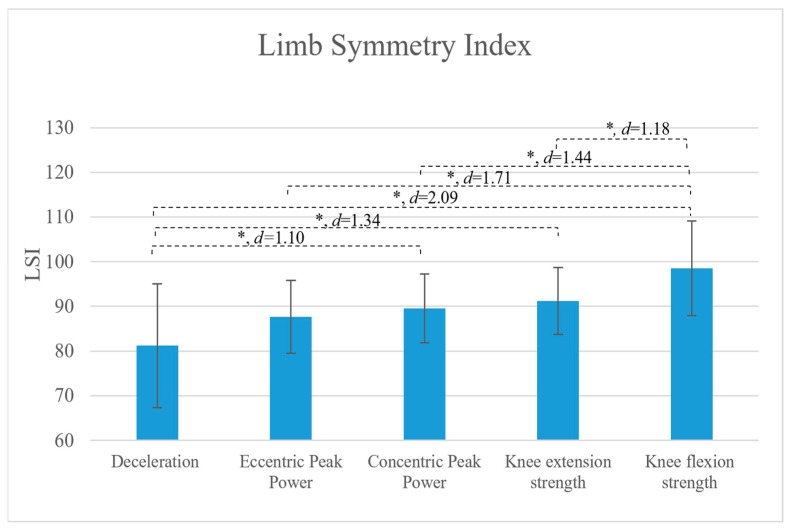
Pairwise comparisons of limb symmetry index across neuromuscular parameters following ACL reconstruction. *-Significant difference *p* ≤ 0.05.

Games-Howell post hoc tests identified significant pairwise differences between several neuromuscular parameters. Cohen’s d effect sizes for significant comparisons are provided (Figure 1). Knee flexor strength LSI was significantly higher than knee extensor strength LSI by 7.28% (*p* = 0.003; moderate effect size). Knee flexor strength LSI also exceeded concentric peak power LSI by 9.00% (*p* < 0.001, large effect size), eccentric peak power LSI by 10.86% (*p* = 0.001, large effect size), and deceleration LSI by 17.32% (*p* = 0.001, large effect size). In addition, deceleration LSI was significantly lower than knee extensor strength LSI by 10.04% (*p* = 0.001, large effect size), and concentric peak power LSI by 8.32% (*p* = 0.007, moderate effect size). No significant difference was found between concentric and eccentric peak power LSIs (mean difference = 1.85%, *p* = 0.81). No statistically significant differences were observed between knee extensor strength and either concentric or eccentric peak power LSIs (mean difference 1.73, 1.66 and *p* = 0.821, *p* = 0.206 respectively).

## 4. Discussion

This study investigated limb symmetry across five neuromuscular parameters in team sport athletes at the return-to-sport testing period after anterior cruciate ligament (ACL) reconstruction. Both strength parameters demonstrated LSI values above the conventional 90% threshold, while power metrics tended to be lower, with concentric peak power approaching this cutoff at 89.5%. Notably, deceleration LSI differed significantly from all concentric measures, and eccentric peak power LSI was significantly lower than strength metrics, confirming our hypothesis that eccentric parameters remain more impaired than concentric ones at this stage of recovery. The findings of this study revealed significantly greater asymmetries in load absorption capacity compared to strength and concentric power measures approximately nine months post-ACL reconstruction, likely indicating incomplete neuromuscular recovery.

A distinctive strength of the present study is the within-subject comparative analysis of limb symmetry indices across multiple neuromuscular parameters, including knee flexor and extensor strength as well as concentric and eccentric power and deceleration metrics during countermovement jumps. Unlike prior research that often examines these or similar metrics separately or contrasts patients with healthy controls [31,32,33], our approach enables direct comparison of the relative magnitude of asymmetries within the same cohort of athletes at a critical stage of recovery. This methodology reveals that eccentric power and deceleration asymmetries are larger than those observed in common strength and power measures, underscoring that residual neuromuscular deficits are more pronounced than what is typically captured by standard return to sport criteria [34]. The findings call into question the sufficiency of current return-to-sport criteria and suggest incorporating eccentric-focused assessments to detect and address these persistent impairments [20,22]. By elucidating the distinct recovery profiles of concentric versus eccentric neuromuscular function, our results provide actionable insights that can guide refinement of return-to-sport decision-making to better reduce re-injury risk and optimize functional outcomes in team sport athletes [3,5].

Our study findings align with the growing body of evidence indicating that limb asymmetries in eccentric metrics often persist long after ACLR, even when clearance for return to sport has been granted based on strength assessments (e.g., limb symmetry index > 90%) [35,36]. Forelli et al. [37] recently demonstrated that at six months post-ACLR, both eccentric and concentric force production remain impaired, with significant interlimb asymmetries. Similar findings were reported by Baumgart et al. [38], who analyzed phase-specific ground reaction forces during bilateral vertical jumps in forty ACL patients at a mean of 2.5 years post-surgery. They found that the ground reaction forces of the operated leg were significantly lower than those of the non-operated leg, predominantly during the eccentric deceleration phase, corroborating our results.

Consistent with these observations, Read et al. [31] demonstrated that eccentric-phase parameters exhibited significant inter-limb deficits in professional soccer players across all stages post-ACLR (<6 months, 6–9 months, and >9 months), with asymmetry values ranging from approximately 6.6% to 17.3% depending on the specific eccentric metric. For example, eccentric deceleration rate of force development asymmetry was notably elevated (up to 17.3% in the <6 months group) and remained substantially high (>9 months post-ACLR group showed ~14.7% asymmetry), highlighting the lasting nature of eccentric loading deficits.

While Read et al.’s study provides valuable insight into persistent eccentric asymmetries at discrete rehabilitation stages, its cross-sectional design limits understanding of the temporal progression of these deficits—a gap effectively addressed by Costley et al., who longitudinally tracked eccentric parameters and torque from six to nine months post-ACLR [39]. The authors investigated amateur multidirectional field sport athletes and reported significant improvements in peak extensor and flexor torque inter-limb symmetry over this period, indicating positive rehabilitation adaptations. However, despite these strength gains, persistent deficits in eccentric function remained evident, particularly during the deceleration and landing phases of vertical jump tasks. However, not all studies report persistent eccentric deficits during ACL rehabilitation. Notably, Kotsifaki et al. [40] found no significant differences in limb symmetry during the eccentric phase of vertical jump tasks between athletes post-ACL reconstruction and healthy controls. This suggests that, in some cohorts, eccentric neuromuscular control may recover to levels comparable to uninjured athletes by return-to-sport stages, highlighting the heterogeneity of recovery profiles.

Although beyond the scope of this study, potential mechanisms for eccentric parameters’ lag behind others should be elaborated. Notably, isokinetic strength testing and jumping performance represent relatively distinct constructs, as evidenced by Menzel et al. [41], who reported that asymmetries observed in these two measures do not necessarily correspond, highlighting their independent nature. Jumping performance, such as that assessed by countermovement jumps (CMJ), relies heavily on complex multi-joint actions and intermuscular coordination [42], with knee angular velocities exceeding 500°/s during the movement [43]. In contrast, isokinetic dynamometry (IKD) assessments commonly used to monitor recovery post-ACLR evaluate isolated joint torque at much slower angular velocities, typically around 60°/s [44]. This fundamental difference suggests that improvements in joint-specific concentric strength may not translate directly to enhancements in dynamic, multi-joint tasks such as jumping. Consequently, training protocols predominantly focused on increasing concentric strength are likely to improve isokinetic strength measures but may have limited carryover to jump performance, which demands rapid force development, intermuscular coordination, and effective force absorption.

Eccentric contractions are biomechanically and neurologically distinct from concentric actions, characterized by active muscle lengthening under load, which requires specialized motor control strategies and greater force modulation to decelerate limb segments effectively [45]. Even explosive concentric training may not adequately stimulate the neuromuscular adaptations necessary for restoring eccentric function because concentric and eccentric contractions involve different recruitment patterns [46] and muscle–tendon unit behaviors [47]. Therefore, without targeted eccentric training emphasizing rapid deceleration and load absorption, eccentric neuromuscular deficits may persist despite adequate concentric strength and power gains [48].

The knee flexor and extensor strength parameters observed in this study largely met the commonly recommended cutoff of 90% limb symmetry [49], indicating that a period of 6–9 months (late-stage recovery) was sufficient to restore knee strength to an acceptable level required for return to play. This aligns with previous research reporting good recovery of thigh strength by six [50] and nine months post-ACL reconstruction in athletes following supervised rehabilitation [51]. Similar findings have also been reported in a recent scoping review within a sample of team sport athletes by van Melick et al. [52], concluding that as soon as within 7 months of rehabilitation, leg strength reached levels comparable to those of healthy athletes, with 9-month LSI reaching 84% and 97% for knee extensors and flexors, respectively. Collectively, study findings suggest that supervised rehabilitation for team sport athletes after ACL surgery enables restoring lower body strength levels, reaching return to sport testing (late-stage recovery).

Concentric peak power asymmetry occupied an intermediate position between strength and deceleration deficits, with an LSI value approaching the conventional cutoff at 89.5%. This finding suggests that although concentric power shows better recovery than eccentric power and deceleration metrics, it may still reflect subtle neuromuscular impairments not captured by isolated strength assessments. Our results align with previous studies demonstrating gradual recovery of concentric power generation following ACL reconstruction, yet emphasize that power measures often lag slightly behind maximal strength gains [9,31]. For example, Read et al. [31] highlighted that countermovement jump power and kinetic asymmetries may persist beyond strength recovery milestones, indicating that power-related deficits often lag behind maximal strength gains during ACL rehabilitation. Moreover, Kotsifaki et al. [40] demonstrated that despite athletes meeting conventional strength symmetry thresholds, asymmetries in countermovement jump performance, including power output, frequently persist at the return-to-sport stage.

This study presents several limitations. First, its cross-sectional design at the return-to-sport phase does not capture longitudinal recovery patterns or causal relationships between neuromuscular deficits and functional outcomes over time. Second, although the limb symmetry index (LSI) quantifies side-to-side differences, it does not account for the possibility of strength or power loss in the uninvolved limb, which may lead to underestimation of overall neuromuscular impairments. Third, the sample included athletes who underwent different types of ACL reconstruction (patellar tendon vs. hamstring tendon autografts), which may influence neuromuscular recovery trajectories and asymmetry patterns, thus introducing variability that was not controlled for. Fourth, the relatively low representation of female athletes limits the generalizability of findings across sexes, especially given potential sex-specific differences in rehabilitation and recovery.

## 5. Conclusions

In conclusion, the current findings indicate more pronounced asymmetries in load absorption (deceleration and eccentric power) than strength and concentric power in team sport athletes at the return-to-sport time frame. From a practical perspective, these findings suggest that standard RTS performance outcomes may overlook critical deficits in load absorption capacity. Incorporating eccentric and deceleration-based assessments into RTS test batteries may enable clinicians and sport practitioners to identify incomplete neuromuscular recovery more accurately. This could lead to better late-stage rehabilitation and a safer return to full sport participation.

## Figures and Tables

**Table 1:** Participant characteristics (mean ± SD), N: number, M: male, F: female, F: football, B: basketball, V: volleyball, H: handball, BPTB: bone patellar tendon bone, HT: hamstring tendon.

Participants	Sport	Age (Years)	Body Height (cm)	Body Mass (kg)	Graft Type	Days Post-Surgery	CMJ Jump Height (cm)
(N = 44, M = 33, F = 11)	(F = 20, B = 10, V = 8, H = 6)	22.5 ± 5.8	180.5 ± 8.38	75.9 ± 13.0	BPTB (n = 33), HT (n = 11)	235.1 ± 55.0	34.5 ± 5.5

**Table 2:** Mean and SD values of selected variables.

Eccentric Peak Power (W)		Deceleration (kg/s)		Concentric Peak Power (W)		Knee Extension 60° (N·m)		Knee Flexion 60° (N·m)	
Injured	Non-injured	Injured	Non-injured	Injured	Non-injured	Injured	Non-injured	Injured	Non-injured
720.48 ± 307.1	815.5 ± 313.2	317.2 ± 114.8	394.7 ± 146.3	1821.2 ± 469.3	2023.4 ± 448.4	204.5 ± 64.3	221.9 ± 61.2	114.7 ± 29.0	118.2 ± 34.5

**Table 3:** Descriptive statistics and Welch ANOVA for LSI across neuromuscular parameters.

Variable	Mean	SD	CI 95%	
			Lower	Upper
Knee extension strength LSI (%)	91.23	7.5	88.96	93.5
Knee flexion strength LSI (%)	98.5	10.6	95.28	101.72
Concentric peak power LSI (%)	89.5	7.7	87.16	91.83
Eccentric peak power LSI (%)	87.64	8.11	85.18	90.11
Deceleration LSI (%)	81.18	13.83	76.98	85.39
One-way ANOVA	F	Df1	Df2	*p*
	12.59	4.0	106.5	<0.001

## Data Availability

Data are available from the author upon reasonable request.
